# How to shorten scientific manuscripts

**DOI:** 10.1002/ece3.11543

**Published:** 2024-06-17

**Authors:** Ayco J. M. Tack, Johan Ehrlén, Tomas Roslin

**Affiliations:** ^1^ Department of Ecology, Environment and Plant Sciences Stockholm University Stockholm Sweden; ^2^ Department of Ecology Swedish University of Agricultural Sciences Uppsala Sweden

**Keywords:** conciseness, revision, scientific writing, shortening, writing guidelines

## Abstract

Many journals have strict word limits, and authors therefore spend considerable time shortening manuscripts. Here, we provide pointers for efficiently doing so while retaining key content. We include general guidance, tips for condensing the different parts of a scientific paper, and advice on what to avoid when shortening manuscripts. We hope that readers will find our guidance helpful.

As young scientists, we thought that article length was restricted due to high printing costs and competition for space. Since then, the length of journal articles appears to have decreased rather than increased—even though nothing is currently printed. This trend may be symptomatic of an age of information overflow, where we need to be succinct to keep the attention of our audience. Alternatively, it may reflect an increased compartmentalization of scientific output, with each paper targeting one or few questions. Whatever the reason, as scientists we should stick to the point and provide a clear and readable message. Succinct writing will help us adhere to the word limits and likely make our paper more read. This article provides guidance on how to do this. So here we go:

## 1 GENERAL TIPS FOR SHORTENING SCIENTIFIC MANUSCRIPTS

### 1.1 Switch to a shortening mindset

Many will be annoyed by having to shorten an existing text. Nonetheless, it is time to switch to a constructive mindset. Most readers will appreciate the same thing said shorter and clearer (Heard, 2022, chapter 20). Consider that in shortening the text, you are not removing content but rather condensing and distilling your text. This makes the essential information and key message stand out more clearly.

### 1.2 Prune the runners

Take a real or digital highlighter. In green, mark those sentences that are short, clear, and crucial for the flow. In yellow, mark sentences and sub‐sentences that are important and cannot be removed in their entirety, but nonetheless seem wordy. In blue, mark sentences that are, after all, not absolutely essential and can be removed. In other words, within each section and sentence, carefully identify the parts that are essential for the reader to follow the storyline. If you have difficulty identifying blue sentences, imagine you can only leave your office after you have identified at least five blue sentences. Or, if you respond better to positive stimuli, imagine that you receive a ball of ice cream for each blue sentence. This exercise will help you focus on the parts of the manuscript that allow for changes.

### 1.3 Avoid repetition

At the manuscript level, do not repeat information given in the Methods section in the Results or Discussion sections. In the discussion, do not reiterate information provided in the Introduction (Heard, 2022). Rather, make sure to clearly reconnect to the questions asked in the Introduction. The discussion needs no lengthy restatement of the findings described in the results, just a short reminder about what results you refer to. You can trust the reader to understand and remember things if they are clearly stated once. At the paragraph level, the same rule holds. While it is important to link the different paragraphs into a coherent story, bringing up a topic in two paragraphs will always take longer than bringing it up once. To check for overlap between paragraphs, you can create a reverse outline, where you distil the key message of each paragraph into a topic sentence accompanied by one or few bullet points (Cayley, 2011; Heard, 2019).

### 1.4 Aim for concise messages

At the level of single sentences, challenge yourself to write succinct messages. For example, rather than first saying that the effect of x on y was significant and then describe the type of effect, you could directly describe the effect. Clarifications at the end of sentences will rarely be needed if the preceding part of the sentence is clear enough.

### 1.5 Delete empty words

Forget the feeling that a statement should sound “scientific”. All readers love simplicity. Delete superfluous adjectives and adverbs, and pompous language. Do we really need to say the effect is “very large,” or that the methods were “beyond the state‐of‐the‐art”? As three additional examples of deleting empty words, a reviewer of the current article suggested to replace “absolutely crucial” with “crucial,” “any lengthy restatement” with “lengthy restatement,” and “bare essentials” with “essentials.” Another way to shorten a manuscript is to use short words instead of long ones, condense wordy phrases and use verbs rather than nouns. For example, our reviewer suggested to replace “as a consequence” with “therefore,” “a bit long relative to the content” with “wordy,” “in many instances, it will even be possible to” with “sometimes you can”, and “is sufficient” with “suffices”. For further guidance and examples, see, for example, Gastel (2015) appendix 2 in Gastel and Day (2022).

### 1.6 Lessen the emotional burden

To ease the pain of ripping up your text, keep an open document for all text you remove. Then, you can rest assured that fine sentences written in the past are not lost forever. Instead, you can return to this treasure trove, or bone heap, in the future (Hancock, 2003). Setting the manuscript aside for a while, and returning to it afresh, can detach you from the text and aid in seeing what can be deleted or condensed.

## 2 SECTION‐BY‐SECTION TIPS FOR SHORTENING SCIENTIFIC MANUSCRIPTS

### 2.1 Rethink the abstract

In switching between journals, you will frequently have to shorten the abstract. Here, it is important to realize that abstracts can be written at different levels of abstraction. To re‐distil the core contents at a higher level of abstraction, find a colleague familiar with the broader (but not narrow) field, and read the abstract of your paper aloud. Your colleague will often be at loss with respect to what you are talking about, and how this work fits into the broader field. Querying your listener will reveal what was clear and what was not. Now try to rework the abstract while focusing on the broad‐brush strokes and what matters to a wide audience. Using a blueprint for the abstract might also be helpful. Most abstracts can be shortened by sticking to a tight format: (i) Use one or two sentences to introduce the study, before you present the questions/hypotheses. (ii) Combine the methods part with the aims—by writing what you aimed to do and how you did it in the same sentence, for example, using the format “*To examine* [aims], *we used* [methods]”. Alternatively, make the methods evident from how you present your result, for example, using the format “[approach or method] *showed that* [finding]”. (iii) Paraphrase the answer to each question in a single sentence. (iv) Use one or two sentences to place the core findings within a broader perspective, like “*Contrary to* [some generally held belief], *we found* [pattern]”.

### 2.2 Focus the introduction

Never say things because you *can*—say them because they are *needed*. Remember that the introduction should provide the background to the questions, expectations for the answers, and insights into why the questions are interesting. By moving from the broad conceptual framework and major gaps toward the specific research questions, you can funnel the reader into your method and Result sections (Gastel, 2016). Consider what broader context you need to make the specific questions relevant and interesting. Importantly, when the reader reaches your aim and questions at the end of the introduction, they should think: “Wow, those are very logical, timely and important questions, I can't wait to see whether the expectations will hold”. Or, if you prefer to end the introduction by revealing the major results, it should be crystal clear how those results fill a major gap within the field. You can simply delete anything that does not provide a background to the key questions. As a tool, return to your highlighter and mark the overarching aim and each of the questions in a separate color. Now mark each piece of the introduction in the respective colors. Remove any sentences that remain unmarked, and check the balance between colors, thus spotting any question receiving an unnecessarily long background. Next, check whether all questions are contributing to the overarching aim. If some do not, remove them throughout the paper. Unless you are writing a review, take care to only cite one or—if you must—two examples to illustrate a point.

### 2.3 Provide only the *relevant* methods

While Methods sections often end up delving into some semi‐arbitrary details, they rarely reach the point where the study is actually reproducible. A short section on methods might then be ideal—when sticking to the point and being accompanied by a detailed supplementary text on the methods. The reader of the main manuscript will then understand what has been done without flipping back and forth between the main text and supplements, *and* have access to all details needed to reproduce the study.

### 2.4 Summarize the results

The Results section should summarize the results and distil their meaning, not describe the raw data. Do not repeat the full set of values presented in a table or a figure. Instead, use the main text to summarize the key messages conveyed by the figure or table. Harder‐to‐interpret statistics and additional relationships belong in the supplement (Heard, 2018). Clearly, in the era of open science, you should share the data—but the Results section is no place to do that.

### 2.5 Focus the discussion

The Discussion section is where you put your results in context. It should focus on the essential, not on every quirk in the results. To shorten the discussion, draw on points 2 and 4 above. What were the key results, and what answers do they provide to your main study questions? What are stray details that you only came to think of because someone had done something remotely similar, allowing you to comment on a slight resemblance or difference? Keep the answers, cut the details. Writing a concise discussion will be easier if you keep in mind what needs to be included. In most cases, a structure with a very brief introductory paragraph summarizing the main findings, one paragraph per question (with your answer), and a concluding paragraph suffices. The question‐specific paragraphs can often be structured as: (i) One sentence stating the answer to the question based on the results, (ii) one or a few sentences giving a feasible explanation for the result, (iii) a few sentences providing a very brief review of similar and contrasting results, and (iv) a concluding sentence. In the concluding paragraph, you can—if you have not already done so—discuss the implications and applications of the findings, and offer ideas for further research. By moving from the answers to the specific research questions to the broad conceptual advances and applications, the reader will instantly grasp how the present work fits into the larger picture (i.e., the inverse funnel; Gastel, 2016).

### 2.6 Identify only the relevant caveats

In writing academic theses, we are encouraged to identify the sources of errors and the things that did not go according to plan. This is good practice and teaches us critical thinking. Nonetheless, what the reader wants to know is what they should now think differently of than before opening this paper. We suggest that you search for your “caveat” sections and re‐evaluate them from the perspective of the now‐shortened story line. What exactly does the reader need to know to interpret your main findings; what conclusion will a potential error affect and how? Relate any caveat to a specific answer (see 5) and spell out how it affects it. If it does not, then cut the caveat. In many cases, it will be shorter to acknowledge the limitations of the approach—and to justify why it was still chosen—in the methods, than to bring it up in the discussion.

### 2.7 Cite only the relevant papers

Just as an introduction needs not review the full field (see 2), the references need not cover all literature published to date. The steps above will already have cut out a great many references. Now finish off by spotting any brackets with more than one or two references, and prune out the less relevant ones.

## 3 WHAT TO AVOID AT ALL COST, EVEN WHEN DESPERATE

### 3.1 Breaking the storyline

Do not shorten the introduction by removing the background for a question, and do not shorten the discussion by leaving some results undiscussed. Readers like short, focused manuscripts, but they dislike broken storylines and loose ends.

### 3.2 Not knowing when to stop condensing

We often think that a short manuscript is one with few words or characters. But those are just metrics that correlate (and not always well) with what really matters: the amount of time and mental effort it takes someone to read and understand the paper (Heard, 2022). If reducing word count helps a reader, then great! But when reducing word count makes reading harder, then it is counterproductive. If you have one of those rare stories that need more space, we recommend to submit to a more length‐liberal journal.

### 3.3 Using abbreviations excessively

In their quest to reduce word counts, many authors introduce their own home‐made abbreviations and acronyms. This increases the cognitive load of the reader (Hughes, 2020). Having to flip back to an earlier section of text where the term was defined (if it even was defined) is a sure recipe for frustration. In contrast, using short, clear and intuitive names for your concepts and variables, where the full explanation is provided only once in the Methods section, is easy on the reader and can save many words.

### 3.4 Fiddling

Fiddling with margins, page breaks, and font size in table and figure legends, will—in contrast to the advice offered above—not result in a better or shorter paper, and frequently act to annoy or waste the time of the editor.

### 3.5 Making your paper more boring

Even when pressed for space, we recommend that you do not remove humorous notes, playful language, thoughtful speculations, originality, and personality (Sand‐Jensen, 2007). To leave a lasting impression, it helps if a paper is enjoyable, engaging, and thought‐provoking.

That's it. Now go and shorten your text.

## AUTHOR CONTRIBUTIONS

**Ayco J. M. Tack:** Conceptualization (equal); writing – original draft (equal). **Tomas Roslin:** Conceptualization (equal); writing – original draft (equal); writing – review and editing (equal). **Johan Ehrlén:** Conceptualization (equal); writing – original draft (equal); writing – review and editing (equal).

## CONFLICT OF INTEREST STATEMENT

We have no conflict of interest to declare.

## Data Availability

No data were used in this paper.
